# The nutrigenetic influence of the interaction between dietary vitamin E and *TXN* and *COMT* gene polymorphisms on waist circumference: a case control study

**DOI:** 10.1186/s12967-015-0652-4

**Published:** 2015-09-02

**Authors:** Maria L. Mansego, Griselda De Marco, Carmen Ivorra, Raúl Lopez-Izquierdo, Sonsoles Morcillo, Gemma Rojo-Martínez, Verónica González-Albert, Fernando Martinez, Federico Soriguer, Juan C. Martín-Escudero, Josep Redon, F. Javier Chaves

**Affiliations:** CIBER Physiopathology of Obesity and Nutrition CIBERobn, CB06/03, Institute of Health Carlos III, c/Sinesio Delgado 6, 28029 Madrid, Spain; Genotyping and Genetic Diagnosis Unit, Fundación de Investigación del Hospital Clínico de Valencia-INCLIVA, Av. Blasco Ibañez 17, 46010 Valencia, Spain; CIBER de Diabetes y Enfermedades Metabólicas Asociadas (CIBERDEM), c/Sinesio Delgado 6, 28029 Madrid, Spain; Cardiovascular Risk Unit, Consorcio, Hospital General, University of Valencia, Av. Tres Cruces 2, 46014 Valencia, Spain; Internal Medicine Unit, Rio Hortega Hospital, c/Dulzaina 2, 47012 Valladolid, Spain; Endocrinology and Nutrition Department, Carlos Haya University Hospital and Instituto de Investigación Biomédica de Málaga (IBIMA), c/Jorge Luis Borges 15, 29010 Málaga, Spain; Hypertension Clinic, Hospital Clínico Universitario de Valencia, Av. Blasco Ibañez 15, 46010 Valencia, Spain

**Keywords:** Vitamin E intake, Oxidative stress, Polymorphism, Waist circumference, Abdominal obesity, Thioredoxin, Catechol-O-methyltransferase

## Abstract

**Background:**

Abdominal obesity (AO) is a common modifiable risk factor for certain non-communicable diseases associated with enhanced oxidative stress (OS). The objective of this work was to investigate whether the interaction between antioxidant vitamin intake and OS-related polymorphisms modulates gene-associated anthropometry in a Spanish population.

**Methods:**

A total of 246 subjects with AO, and 492 age and gender matched non-AO subjects were included in the study. Anthropometric, biochemical, and OS parameters, and antioxidant dietary intake data were assessed using validated procedures. DNA from white blood cells was isolated and the genotype of seven polymorphisms from genes involved in OS (pro-oxidant and antioxidant) were analyzed using the SNPlex system. The effects of the c.-793T > C polymorphism on promoter activity and thus thioredoxin (*TXN*) activity were examined using reporter assays.

**Results:**

The AO group had higher 8-Oxo-2′-deoxyguanosine levels and took in less vitamin A and vitamin E compared to the non-AO group. Logistic regression analysis revealed that the rs2301241 polymorphism in *TXN* and rs740603 in catechol-O-methyltransferase (*COMT)* were associated with waist circumference (WC) and AO. Moreover, these polymorphisms were more strongly associated with variations in WC in subjects with low vitamin E intakes. A promoter assay revealed that the T to C conversion at c.-793 (rs2301241) induced a more than two fold increase in reporter gene expression.

**Conclusions:**

WC is associated both with dietary vitamin E intake and genetic variants of *TXN* and *COMT* suggesting that existence of a complex nutrigenetic pathway that involves regulation of AO.

**Electronic supplementary material:**

The online version of this article (doi:10.1186/s12967-015-0652-4) contains supplementary material, which is available to authorized users.

## Background

The increasing number of overweight and obese people (with a body mass index (BMI) of 25 or more) is a well-recognized public health problem in Europe and in other developed countries [1]; in Spain this group accounts for an estimated 60 % of the population, although the prevalence varies among European regions [2, 3]. Moreover, the prevalence of abdominal obesity (AO), as estimated independently of BMI by using waist circumference (WC) as a proxy, varied between 3.8 and 33.2 % in the studied countries, and is about 20 % in Spain [4]. AO is known to increase the risk of particular diseases, particularly type 2 diabetes, resulting in a greater risk of death [5, 6].

Factors related to the development of visceral adiposity are complex and multiple mechanisms have been implicated [7], although one of these mechanisms, increased oxidative stress (OS), is now being more intensively studied [8, 9]. The impact of increased quantities of reactive oxygen species (ROS) on several cellular systems in patients with AO has recently been described as a contributing factor not only to systemic OS but also to the overall fat storage pattern [10]. In fact, ROS in adipocyte cells produce alterations in the endoplasmic reticulum which increases the proportion of unfolded or misfolded proteins and thus also cellular stress [11]. Furthermore, they allow long-chain fatty acids bound to coenzyme A to accumulate, which lowers mitochondrial activity and encourages fat storage. Likewise, OS increases insulin resistance which further contributes to fat accumulation in muscle, liver, and adipose tissue [12].

The level of OS depends on the interplay of both enzymatic and non-enzymatic pro-oxidant and antioxidant mechanisms, and dietary antioxidant intake seems to have an important role. Hence, low intake levels of the main antioxidant vitamins (vitamins A, C, and E) have been associated with undesirable anthropometric parameters such as increased BMI and waist perimeter. In addition, antioxidant diets can modulate the generation of both ROS and antioxidant molecules, which are partly controlled by genes coding for proteins in different systems [13, 14]. Vitamin E has been described to moderately improve OS status and glucose metabolism in an animal model of diet-induced obesity [15] and chronic dietary vitamin A supplementation regulates obesity in obese-phenotype rats, possibly by upregulating the uncoupling protein 1 (*UCP1*) gene thus causing adipose tissue loss [16].

Certain single-nucleotide polymorphisms (SNPs) in antioxidant enzymes (including superoxide dismutase (*SOD*), glutathione peroxidase (*GPX*), catalase (*CAT*), or thioredoxin (*TXN*)) or in oxidant enzymes (such as catechol-O-methyltransferase (*COMT*)) may lead to decreased or impaired enzymatic activity regulation thereby promoting OS [17]. There is also evidence that diet can interact with genetic variation, causing inflammation and dyslipidemia [18, 19]. Interestingly, *CAT* alleles with the T-20C (rs1049982) SNP are associated with improved recovery from malnutrition in elderly patients, therefore suggesting that high-risk subpopulations could be identified by genotyping [20]. However, there is scarce information available regarding the effect of antioxidant intake and the influence of OS-related gene polymorphisms on anthropometric parameters. Therefore, the aim of the present study was to investigate whether the interaction between antioxidant vitamin (vitamins A, C, and E) intake and OS-related polymorphisms modulates gene-associated anthropometry in a Spanish population.

## Methods

### Subjects

We performed a case-controlled trial on a selected subsample of age and sex matched AO and non-AO individuals (1:2 ratio) from the Hortega study, a population-based study in Valladolid, Spain [21]. All the subjects included in the study were Caucasian and lived in an area with a low immigration rate. Four hundred and ninety-two control subjects were selected and compared to 246 subjects with AO according to the ATP-III criteria [22] (defined as an estimated WC of more than 102 cm in men or 88 cm or more in women). Inclusion criteria were: Caucasians older than 18 years old with no serious concomitant disease (epilepsy, psychosis, alcoholism, lung cancer, chronic cardiopulmonary heart disease, pulmonary emphysema, or terminal disease with a life expectancy of less than three months) or psychiatric disorders which could interfere with the study. All the procedures followed were in accordance with the 1975 Helsinki Declaration, as revised in 2000 [23], and the standards of the local ethical committee for human experimentation (at the Clinic Hospital in Valencia, Spain, signed off on 28 April 2011). Informed written consent was given by all patients included in the study.

### Demographic characteristics, anthropometry, and biochemical assessments

Demographic characteristics included the age, sex, and education level of the subjects. The latter was grouped into two categories for analysis: less than high school or greater than or equal to high school. BMI was calculated as the ratio between weight (kg) and squared height (m^2^). Weight was measured using precise scales (without shoes and with patients wearing only light clothing) and height was determined in a similar way. WC was measured at the mid-level between the lower rib margin and the iliac crest, and hip circumference was measured at the point of maximum buttock extension; the waist-hip ratio (WHR) was calculated as WC divided by hip circumference. All measurements were recorded by trained investigators or nursing personnel. The inter-observer error rate was less than 2 cm.

After a mean of 3 h fasting, venous blood samples were collected into k3-EDTA tubes for hematological and biochemical profiling using extracted nucleated-cell DNA. Basic serum biochemistry and lipid profiles (total cholesterol, high-density lipoprotein [HDL] cholesterol, and triglycerides) were assessed with a Hitachi 917 auto-analyzer (Boehringer, Germany). Glucose was measured using the glucose-oxidase method and low-density lipoprotein (LDL) cholesterol was calculated using the Friedewald formula. Metabolic syndrome was defined by ATPIII criteria [24].

### Oxidative stress parameters

Oxidative stress markers were measured in blood plasma. DNA damage, assessed by the formation of 8-oxo-deoxyguanosine (8-OXO-DG) was quantified by high-performance liquid chromatography (HPLC) electrochemical (EC) detection after complete enzymatic digestion [25]. Malondialdehyde (MDA), an indicator of o-lipid oxidation, was analyzed by HPLC [26]. Protein content was measured using the Bradford method [27]. The glutathione system is the most important free-radical scavenger, therefore we measured the ratio of oxidized glutathione (GSSG) and reduced glutathione (GSH) as an estimation of the overall level of OS; GSSG was analyzed by HPLC and GSH was analyzed using the glutathione-S-transferase assay [28].

### Nutritional intake assessment

The Mediterranean diet is the typical diet for the geographical location of the population examined in this study. This typically includes proportionally high consumption of olive oil, legumes, unrefined cereals, fruits, and vegetables, moderate to high consumption of fish, moderate consumption of dairy products (mostly as cheese and yogurt) and wine, and low consumption meat and meat products [29]. In this study 24 h recall food-intake data were recorded on three different days (once during the interview, and then 4 and 8 weeks later, which were sent by the patient by mail). Experienced dietitians carried out the interview using the previously validated semi-quantitative Food Frequency Questionnaire (FFQ) to determine the number of portions of each food type consumed monthly; photographs were also used to determinate portion sizes. These two independent methods of diet evaluation were averaged and the data was further analyzed as described below.

The dietary nutrient composition was determined at the Institute of Nutrition and Food Technology at the University of Granada using food composition tables and was analyzed with the previously-validated [30] *Alimentación y Salud* (Food and Health) software program, version 0689.01 (BitASDE General Médica Farmacéutica, Valencia, Spain) designed by the same institute. Vitamin A intake (in µg/day) and vitamin E and vitamin C intake (in mg/day) were estimated and all the participants were assigned into one of two groups based on their median vitamin E intake: “low’’ (9.0 mg/day or less) and ‘‘high’’ (more than 9.0 mg/day).

### DNA isolation and single-nucleotide polymorphism selection and genotyping

DNA was isolated from peripheral blood cells using the Chemagic system (Chemagen); its quality was assessed using the PicoGreen dsDNA Quantitation Reagent (Invitrogen, Carlsbad, CA) and it was diluted to a final concentration of 100 ng/μl. Seven oxidative-stress related SNPs from six genes were selected based on their minor allele frequency (MAF) in Caucasians (0.1 or more), location, distribution, tag-SNP characteristics using the SySNP program (http://www.sysnps.org/‎), and their possible effects on function as determined in previous studies by our group [31]. The selected genes and reference names, chromosome position, locus, and the MAF of the selected SNPs are shown in Table 1. The selected SNPs were genotyped using a genotyping system based on an oligo-ligation assay/polymerase chain reaction technology (SNPlex, Applied Biosystems, Foster City) following the manufacturer’s guidelines. The nomenclature of the polymorphisms is based on recommendations by den Dunnen and Antonaraki [32].

**Table 1:** Characteristics of selected polymorphisms

HGN	Gene name	dbSNP^a^	Tag-SNP^b^	Chr position	Reference^c^	MAF^d^
*CAT*	Catalase	rs1049982	Y	11:34417117	c.-20C > T	0.36
*SOD3*	Superoxidedismutase 3	rs2536512	Y	4:24410413	c.172G > A (p.A58T)	0.37
*GPX1*	Glutathione peroxidase 1	rs3448	Y	3:49371755	c.*891C > T	0.25
*TXN*	Thioredoxin	rs4135168	Y	9:112056706	c.24 + 1807G > A	0.12
		rs2301241	Y	9:112059329	c.-793T > C	0.48
*XDH*	Xanthine dehydrogenase	rs17323225		2:31446769	c.1936T > C (p.I646V)	0.06
*COMT*	Catechol-O-methyltransferase	rs740603	Y	22:19945177	c.-91-3545G > A	0.39

*HGN* HUGO gene nomenclature, *Chr* chromosome

^a^NCBI dbSNP Build 126

^b^Tag-SNP by HapMap in Caucasoid subjects (*Y* yes)

^c^Begins in the first nucleotide 5′ of the ATG start codon, Build 126, Ensembl release 41 as suggested by the Human Gene Nomenclature Committee (HGNC)

^d^MAF (minor allele frequency), data obtained from 1000 genomes

### Measuring thioredoxin promoter activity

To construct a wild-type *TXN* reporter plasmid a 969-bp fragment of the human *TXN* promoter (nucleotides −952 to +17 relative to the translation initiation site) was amplified with the following primers: (forward) 5-CGGGGTACCATCAGTCCCTGCAAGACACC-3 and (reverse) 5-GGAAGATCTTCGATCTGCTTCACCATCTTGGC-3 from homozygote DNA samples with the rs2301241 (c.-793T > C) polymorphism. Reporter vectors containing the c.-793T > C variation were produced by oligonucleotide-based mutagenesis of the wild-type reporter plasmid. PCR products (wild type and variant) were cloned into KpnI/BglII sites on the pGL3 basic vector (Promega). The sequence and integrity of each construct were confirmed by dideoxynucleotide-based sequencing.

Promoter assays were carried out by first growing HuH-7 cells in Dulbecco’s Modified Eagle’s medium containing 25 mM glucose, supplemented with 10 % fetal bovine serum and antibiotics (100 U/ml penicillin and 100 µg/ml streptomycin) at 37º C in a humidified atmosphere with 5 % CO_2_. Plasmid transfections were performed on attached cells at 50 % confluence. Cells were transfected using FuGENE^®^HD Transfection Reagent (Roche Applied Science) with 1 mg of pGL3-TXN reporter constructs (wild type or mutated) and 50 ng of a promoter less renilla luciferase construct (pRL-0). Transactivation activity was measured 24 h after transfection in a Wallac 1420 VICTOR2 luminometer using the dual luciferase reporter assay system (Promega) following the manufacturer’s instructions. Relative light units were determined by quantifying the signal from firefly luciferase after normalizing it against co-transfected renilla luciferase activity in the same sample. Finally, these relative values were normalized against a mock transfection experiment. Each expression construct was transfected in triplicate wells and the experiments were repeated three times.

### Measurement of plasma thioredoxin levels

The plasma level of TXN was measured using an enzyme-linked immunosorbent assay kit following the manufacturer’s instructions (Cusabio Biotech Co., Ltd) in 88 subjects with the rs2301241 SNP homozygote genotype (TT or CC). Samples were placed into micro-wells pre-coated with a human-specific antibody (HRP-conjugate) and successively with one substrate and a chromogenic system. The reaction was stopped and the absorbance was read at 450 nm.

### Statistical analysis

Data are expressed as the mean (±the standard deviation, *SD*) except when otherwise indicated. The Hardy–Weinberg equilibrium was tested using the Chi square (χ^2^) test for each polymorphism. The participant characteristics were assessed as a function of AO. The differences between the mean and *SD* for each variable were assessed by one-way analysis of variance (ANOVA), and were compared with contingency tables and using the χ^2^ test. The association between polymorphisms and anthropometric parameters (BMI, WC, and hip and WHR) and OS were examined using analysis of covariance (ANCOVA), focusing on the dominant model. The association of AO with each polymorphism was analyzed by logistic regression, adjusting the model for the confounding variables of age, gender, education level, hypertension, glucose, HDL-cholesterol, triglycerides, 8-OXO-DG, and alcohol consumption, except as otherwise indicated.

To identify gene–gene interactions we implemented a genetic, model-free, non-parametric multifactor dimensionality reduction (MDR) strategy [33] using 100-fold cross validation and 1000-fold permutation testing. We used the MDR software (version 3.0.2) which is freely available (http://www.epistasis.org). MDR results were considered statistically significant at the *p* = 0.05 level. We used ANOVA to study the interaction between diet, vitamins A and E, the WC-associated SNPs (rs2301241 for the *TXN* genotype or rs740603 for the *COMT* genotype), and WC, using the same covariate factors mentioned above.

Pearson correlations were fitted to evaluate any potential correlation between OS parameters and anthropometric measurements, metabolic features, and dietary factors. Statistical analyses were performed with SPSS 19.0 software (SPSS Inc., Chicago, IL, USA). A *P* value of less than 0.05 was considered to be statistically significant, except for the genotype association analysis where we used the Bonferroni correction: 0.05/(number of SNPs; 7) giving statistical significance at *p* value <0.007.

## Results

### General study population characteristics

A total of 738 participants, 390 (52.8 %) men, were suitable for the analysis. The main study population characteristics, grouped by AO, are presented in Table 2. A total of 246 AO and 492 non-AO individuals were included. As expected, participants allocated to the AO group had a higher BMI, WC, and hip and WHR than the non-AO group. There were also significant differences in the levels of glucose, triglycerides, hypertension, metabolic syndrome, and alcohol consumption between AO-classification groups; in addition, HDL-cholesterol was generally higher and education levels poorer in the AO group.

**Table 2:** General characteristics of the study population, categorized by abdominal obesity

Variables	Non-AO (n = 492)	AO (n = 246)	*p* value
Age (years)	61 (17)	61 (17)	0.972
Gender (M/F)	260/232	130/116	1.000
Education level (<high school (%))	291 (59 %)	164 (67 %)	0.045
BMI (kg/m^2^)	26 (3)	30 (3)	<0.001
WC (cm)	87 (10)	103 (9)	<0.001
Hip (cm)	101 (6)	109 (8)	<0.001
WHR	0.86 (0.09)	0.95 (0.09)	<0.001
Hypertension (%)	190 (39 %)	128 (52 %)	<0.001
Glucose (mg/dl)	91 (13)	95 (16)	<0.001
Total cholesterol (mg/dl)	205 (35)	204 (39)	0.763
HDL-cholesterol (mg/dl)	52 (14)	46 (12)	<0.001
Triglycerides (mg/dl)	168 (82)	207 (89)	<0.001
Dyslipidemia (%)	82 (17 %)	52 (21 %)	0.084
Diabetes (%)	40 (8 %)	29 (12 %)	0.110
Metabolic syndrome (%)	112 (23 %)	195 (79 %)	<0.001
Smokers (%)	96 (20 %)	51 (21 %)	0.386
Alcohol consumption (g/day)	6.4 (8.6)	9.7 (13.1)	<0.001
8-OXO-DG	3.1 (1.6)	3.4 (1.7)	0.016
MDA (μmol/mg protein)	0.59 (0.54)	0.64 (0.61)	0.289
GSH/GSSG	4.4 (2.5)	4.2 (2.6)	0.334
Vitamin A intake (µg/day)	887 (519)	772 (504)	0.009
Vitamin C intake (mg/day)	225 (137)	207 (138)	0.108
Vitamin E intake (mg/day)	10.0 (4.6)	8.7 (4.4)	<0.001

Data are mean (±SD). Education level was grouped into two categories for the analysis as less than high school (<high school) and greater than or equal high school. The value of 8-OXO-DG was expressed as the number of oxidized bases/10^6^ deoxyguanosine. *P* values denote differences between non-AO group and AO group

*Non-AO* non-abdominal obesity group, *AO* abdominal obesity group, *BMI* body mass index, *WC* waist circumference, *WHR* waist hip ratio, *SBP* systolic blood pressure, *DBP* diastolic blood pressure, *HDL* high-density lipoprotein, *8-OXO-DG* 8-oxo-deoxyguanosine, *MDA* malondialdehyde, *GSH/GSSG* ratio between reduced and oxidized glutathione

### Oxidative stress and antioxidant dietary intake

OS parameter concentrations (8-OXO-DG, MDA, and the GSSG/GSH ratio) and dietary antioxidant (vitamins A, C, and E) intake, categorized by the presence or absence of AO are shown in Table 2. 8-OXO-DG levels were higher and vitamin A and E intake was significantly lower in AO patients than in non-AO participants.

### Single-nucleotide polymorphisms, oxidative stress parameters, anthropometry and abdominal obesity

The Hardy–Weinberg equilibrium was maintained for all SNPs in the study population except for rs17323225 that was excluded from subsequent analysis. No significant differences were observed for OS parameters according to the selected SNP genotypes (data not shown). The *TXN* rs2301241 and the *COMT* rs740603 polymorphisms were significantly associated with WC after Bonferroni correction (*p* < 0.007; Table 3), and likewise, these two SNPs were associated with AO [TT vs. TC+CC rs2301241 genotypes, odds ratio [OR] = 0.70 [95 % confidence interval (CI) 0.50–0.97, *p* = 0.034] and GG vs. GA + AA genotypes for the rs740603 *COMT* polymorphism, OR = 0.73 (95 % CI 0.53–0.99, *p* = 0.045)]. There were no significant differences between the genders (Additional file 1: Table S1). To rule out the influence of other comorbidities associated with AO, we analyzed these two SNPs in patients with or without metabolic syndrome, but did not observe any significant association (Additional file 1: Table S2). Moreover, we categorized metabolic syndrome-related characteristics in the study population by the *TXN* and *COMT* SNP genotype, but found that only age differentially-associated depending on the genotype of each SNP (Additional file 1: Table S3). Statistical correlation analysis between biochemical and anthropometric parameters in AO and non-AO groups and according to these genotypes (Additional file 1: Table S4) showed that WC is only positively correlated with glucose in TT and GG carriers of *TXN* and *COMT* genes, respectively, in the non-AO group.

**Table 3:** Selected SNP genotyping distribution of anthropometry by dominant genetic model

HGN	SNP name	Genotype	N	WC (cm)	BMI	Hip (cm)	WHR
*CAT*	rs1049982	CC	264	92.2 ± 0.4	27.0 ± 0.1	104.6 ± 0.8	0.90 ± 0.01
	HWE: 0.17	CT+TT	322	92.5 ± 0.3	27.4 ± 0.1	105.3 ± 0.3	0.91 ± 0.00
	MAF T: 0.34			0.622	0.058	0.415	0.173
*SOD3*	rs2536512	AA	250	92.4 ± 0.4	27.2 ± 0.1	105.4 ± 0.8	0.91 ± 0.01
	HWE: 0.23	AG+GG	335	92.3 ± 0.3	27.3 ± 0.1	105.2 ± 0.3	0.91 ± 0.00
	MAF G:0.36			0.811	0.459	0.784	0.945
*GPX1*	rs3448	CC	340	92.1 ± 0.3	27.2 ± 0.1	106.8 ± 1.0	0.92 ± 0.01
	HWE: 0.11	CT+TT	245	92.7 ± 0.4	27.3 ± 0.1	105.1 ± 0.3	0.91 ± 0.00
	MAF T: 0.25			0.242	0.859	0.093	0.366
*TXN*	rs4135168	AA	328	91.9 ± 0.3	27.3 ± 0.1	105.8 ± 0.4	0.92 ± 0.00
	HWE: 0.61	AG+GG	246	92.9 ± 0.4	27.2 ± 0.1	104.5 ± 0.4	0.91 ± 0.00
	MAF G:0.24			0.031	0.824	*0.021*	0.277
*TXN*	rs2301241	TT	172	93.4 ± 0.4	27.1 ± 0.2	106.3 ± 0.5	0.91 ± 0.01
	HWE: 0.24	TC+CC	415	91.9 ± 0.3	27.3 ± 0.1	104.7 ± 0.3	0.91 ± 0.00
	MAF C:0.47			0.004	0.322	*0.01*	0.847
*COMT*	rs740603	GG	225	93.3 ± 0.4	27.0 ± 0.2	105.7 ± 0.4	0.92 ± 0.00
	HWE: 0.75	GA+AA	355	91.6 ± 0.3	27.4 ± 0.1	104.9 ± 0.4	0.91 ± 0.00
	MAF A:0.38			0.001	0.088	0.159	0.139

Values are the mean ± SEM. Differences in the mean values were assessed by ANOVA and adjusted for age, gender, education level (< high school), hypertension, glucose, high-density lipoprotein cholesterol, triglycerides, 8-oxodeoxyguanosine, and alcohol consumption. The values in italics are statistically significant (*p* < 0.05) and the values in italic and bold are statistically significant after Bonferroni correction (*p* < 0.007). *P* values denote differences between genotypes using a dominant genetic model for all of the SNPs

*HGN* human gene nomenclature, *N* number of individuals for each genotype or genotype group, *HWE* Hardy–Weinberg equilibrium, *MAF* minor allele frequency, *WC* waist circumference

**Fig. 1:**
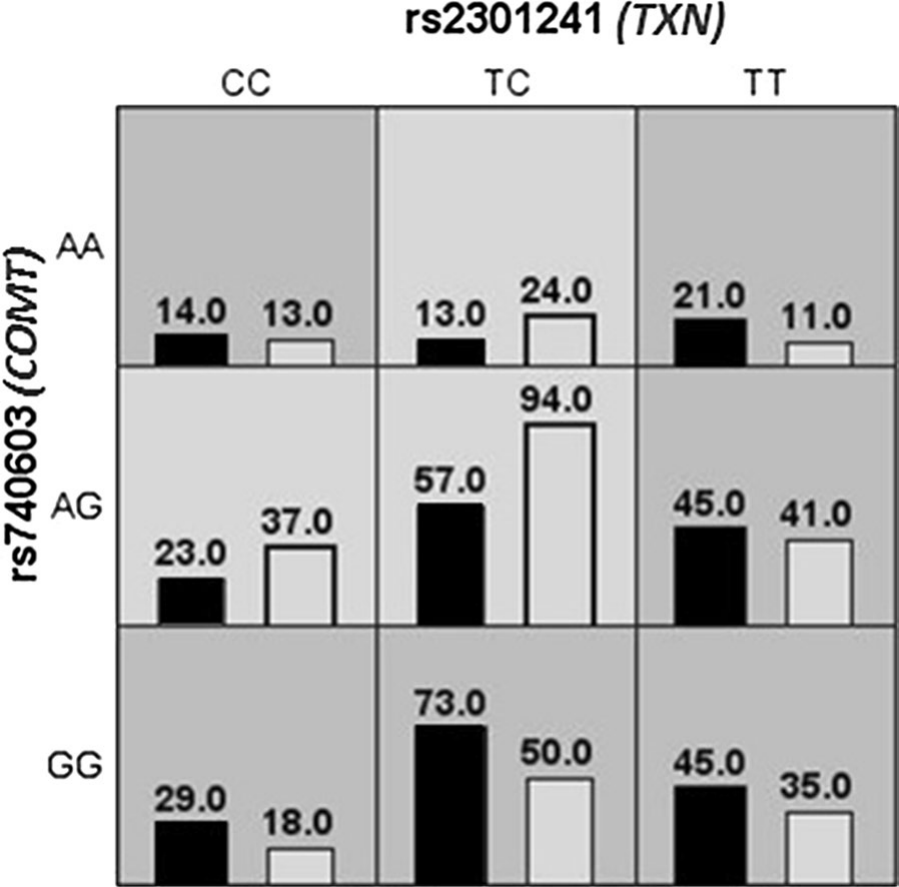
Summary of multi-factor dimensionality (MDR). The results of MDR for two-locus genotype combinations associated with abdominal obesity, as estimated by waist circumference. The distribution of abdominal obesity cases (*left bar*s) and non-abdominal obesity controls (*right bars*) for each genotype combination are shown. The cells shaded in *dark grey* are considered to be “high risk” and the cells shaded in *light grey* are considered “low risk”

Figure 1 summarizes the best interaction models we obtained from the MDR analysis. The best two-locus model for predicting AO was rs2301241 (*TXN*) and rs740603 (*COMT*) with an improved testing-accuracy of 57 % (cross-validation consistency, 100; permutation *p* value < 0.001). After identifying the high-risk allele combinations by MDR analysis, we applied a logistic regression to calculate the their possible effects, observing that the combination of the rs2301241 and rs740603 A and C alleles respectively, was associated with lower WCs [AC vs. TG allele combination, difference = −2.52 (95 % CI −3.89 to −1.15, *p* = 0.00033)] and AO [OR = 0.64 (95 % CI 0.46–0.89, *p* = 0.0087)].

### Interaction between waist circumference, vitamin E intake, and single-nucleotide polymorphisms

**Fig. 2:**
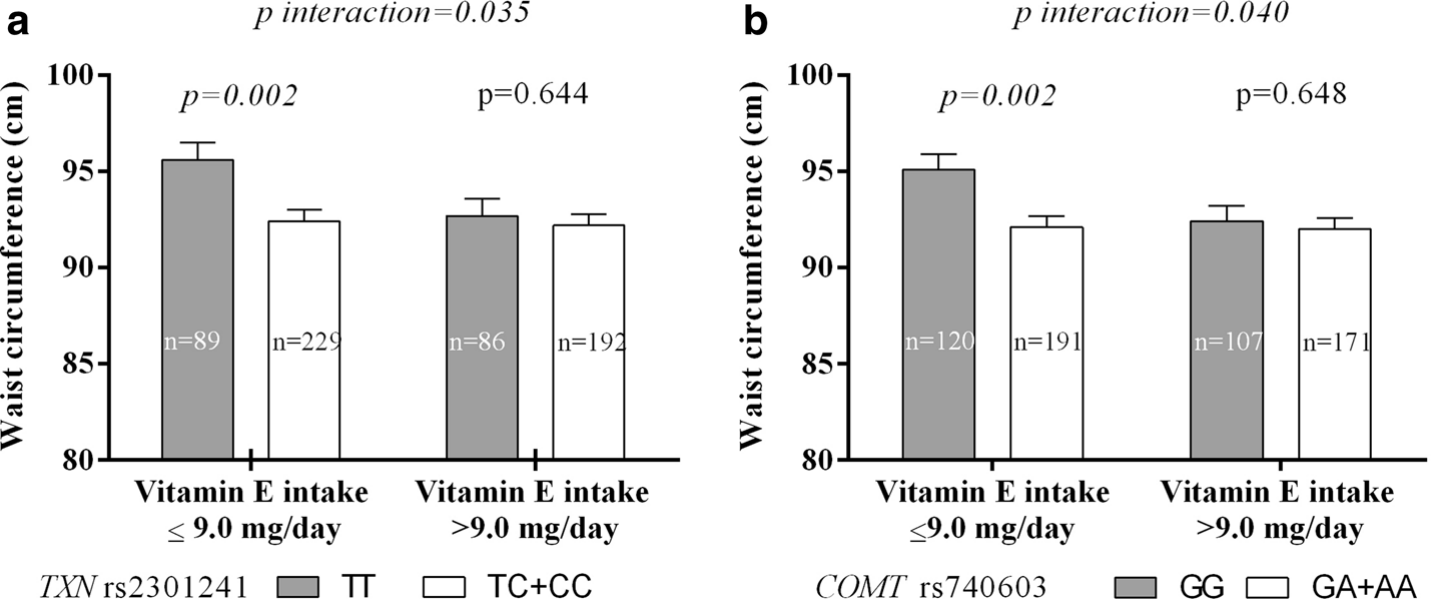
Analysis of the interaction between rs2301241 (**a**), rs740603 (**b**), and vitamin E intake on waist circumference. Data are the mean (±SEM). *P* values were assessed by one-way analysis of variance (ANOVA) and adjusted by age, gender, education level (<high school), hypertension, glucose, high-density lipoprotein cholesterol, triglycerides, 8-oxodeoxyguanosine, and alcohol consumption

With regard to rs2301241 (*TXN*), the TT-genotype subjects that consumed diets low in vitamin E had higher WCs than the C-allele carriers (Fig. 2a); the mean differences between these two genotypes were significantly different in this group (3.16 cm; *p* = 0.002, using a dominant [TC or CC] model). On the other hand, no significant genotype-based differences were found among subjects who consumed diets high in vitamin E. Carriers of the rs740603 (*COMT*) A allele who consumed diets low in vitamin E had a lower WC than homozygotes for the G allele (92.0 ± 12.1 vs. 95.12 ± 13.14 cm, *p* = 0.002; Fig. 2b), whereas WC was higher in GG genotype carriers who consumed diets low vitamin E compared to those with diets high in vitamin E (95.12 ± 13.14 vs. 92.4 ± 11.2 cm, *p* = 0.016). The interaction between SNPs and vitamin E intake was found to significantly influence WC in a dominant [GA or AA] model (*p* = 0.040). There were no significant interactions between vitamin A intake and SNPs (data no shown).

### Molecular and functional studies in the thioredoxin genetic variant

**Fig. 3:**
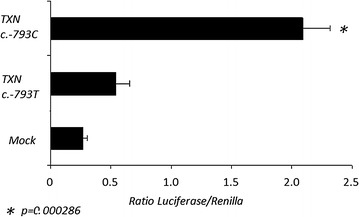
Analysis of transcriptional activity in the −952 to +17 region of human *TXN* promoter. HuH-7 cells were transiently transfected with 1 mg of luciferase reporter constructs and 50 ng of a promoter less renilla plasmid. Luciferase activity was normalized against the values of the renilla internal control. The data represent mean ± SEM (*n* = 3) and are expressed as fold-induction with respect to the empty vector (pGL3basic). *p < 0.005 with the *TXN* c.-793T and *TXN* c.-793C constructs

We investigated possible mechanisms by which the c.-793T > C promoter variation (rs2301241) affects *TXN* transcription activity. Genetic variations in the promoter region can influence gene expression by affecting mRNA transcription. Therefore, reporter constructs containing the rs2301241 SNP promoter in the *TXN* gene were prepared, and their transcriptional activities were measured in HuH-7 human hepatocyte cells. When compared with the standard c.-793T allele, the transcriptional activity of the c.-793C variant allele was up to four times higher in these cells (Fig. 3). A computational search for DNA-binding proteins using Genomatix software [34] revealed that heat shock factor 2 (HSF2) can bind to this reverse region [gtcctgacctcctGAAGtctcagtg] only when c.-793C allele is present in the *TXN* promoter.

**Fig. 4:**
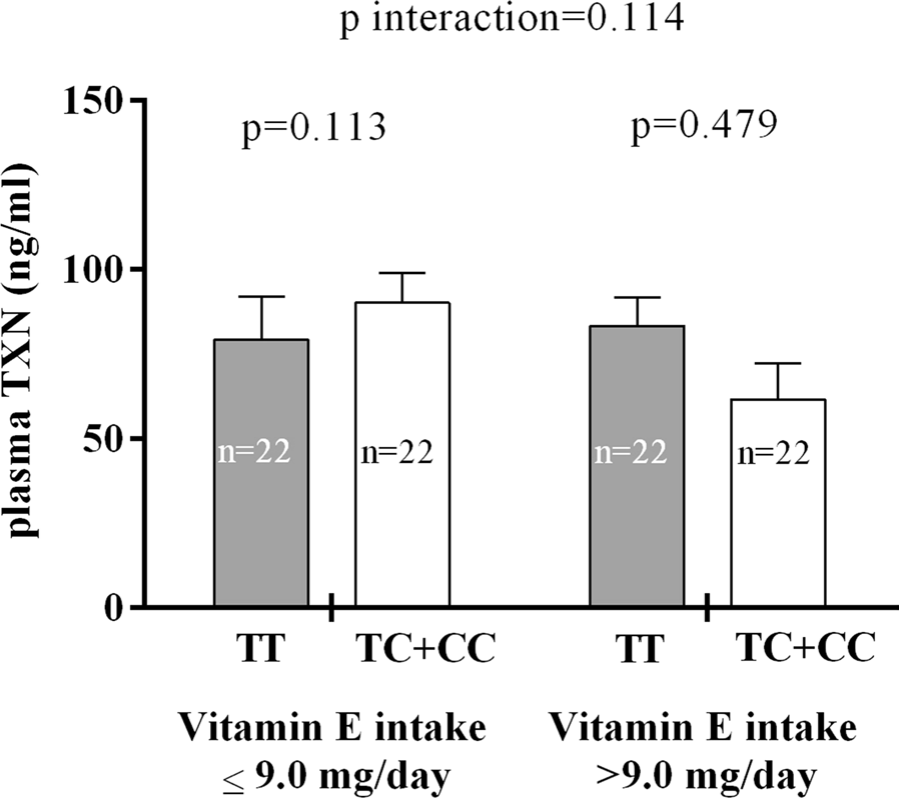
Analysis of the interaction between rs2301241 and vitamin E intake on plasma TXN levels. Data shown are the mean (±SEM). *p* (*p* value) was assessed by ANOVA and adjusted by age, gender, education level (<high school), hypertension, glucose, high-density lipoprotein cholesterol, triglycerides, 8-oxodeoxyguanosine, and alcohol consumption

The plasma TXN level was measured in a subset of 88 individuals selected according to their rs2301241 genotypes and was found to be directly related to WC (r = 0.596, *p* < 0.001). Although no differences in plasma TXN levels between rs2301241 polymorphism genotypes were found, there was a trend toward significance for the interaction between rs2301241, plasma TXN levels, and vitamin E intake (*p* = 0.114), with higher levels of plasma TXN present in C-allele individuals who consumed diets low in vitamin E (Fig. 4).

## Discussion

In our study population the rs2301241 (*TXN* gene) and rs740603 (*COMT* gene) polymorphisms were associated with WC in low vitamin E intake individuals. Moreover, a significant gene–gene interaction was found between these two SNPs, WC, and AO, thus providing the first evidence that the *TXN* and *COMT* gene variations modulate the effect of dietary vitamin E on WC. In this population, AO was associated with an increase in 8-OXO-DG and with a decrease in vitamin A and vitamin E intake. These data concur with recent studies suggesting that systemic OS correlates with obesity, BMI, and fat mass [35]. It is known that OS can increase adipocyte hypertrophy, cellular stress, cytokine and adipokine secretion, and the degradation of many proteins involved in metabolic regulation [11]. In turn, low vitamin A and E intake were associated with AO, and there was also a trend toward a significant association with low vitamin C intake, as previously reported by other authors [14]. In fact, recent evidence suggests that some micronutrient deficiencies are related to obesity and fat deposition: specifically, vitamin A is a known regulator of adipose tissue growth [36].

AO is believed to be a greater risk factor for cardiovascular health than for obesity. Giving weight to this idea, our finding that there was a significant association between the studied polymorphisms and WC but not BMI supports the notion that weight and fat distribution are regulated by different genes. In this study we only found an association with WC in two of the seven SNPs we studied: there was no association with the *CAT*, *SOD*, or *GPX1* SNPs and anthropometry suggests the ROS/OS pathways may not be directly related to WC via these genes. Although these enzymes have been linked to some types of obesity or metabolic syndrome [37] we did not find any significant associations between these SNPs and different anthropometric parameters.

However, the *TXN* and *COMT* genes were related to OS mechanisms that have been associated with WC. The protein encoded by *TXN* acts as a homodimer and is involved in many redox reactions. For example, a novel interaction between upregulated TXN-TXNRD1-system mediated OS defense mechanisms and downregulated angiogenesis pathways was recently described as an adaptive response in obese subjects [38]. In addition, this antioxidant protein has been related to glucose metabolism in adipocytes and in other tissues [39–41]. Interestingly, we showed that vitamin E can protect against increases in WC in carriers of the rs2301241 (*TXN*) T allele and that this group tends to maintain plasma TXN levels similar to C allele carriers. Similarly, England et al. (2012) reported that the effect of α-tocopherol supplementation on the production of inflammatory cytokines appears to be dependent on SNP genotypes in genes involved in inflammation or responses to OS in healthy volunteers [42]. These genotype-specific differences may help explain some of the discordant results in studies that used vitamin E supplementation.

The protein encoded by *COMT* catalyzes the transfer of a methyl group from S-adenosyl methionine to catecholamines, including the neurotransmitters dopamine, epinephrine, and norepinephrine [43]. In addition, the influence of catecholamines and sex steroids on food intake/satiety [44] and metabolism [45] is well known. A recent study has found that carriers of the *COMT* polymorphism valine allele (V158M) tend to have a higher BMI than those with the Met158 allele [46]. In our population, we found a protector polymorphism (rs740603) which was related to WC and vitamin E intake. This variation is a tag-SNP located in an intronic region of *COMT*. Furthermore Ittiwut et al. (2011) reported that this polymorphism has potentially-functional variants which are related to a psychiatric phenotype [47]. Concerning the interaction between vitamin E and *COMT*, our findings show that the GG homozygote subjects who consumed a low vitamin E diet had significantly higher WCs than those carrying the A allele. Thus, these results are consistent with the well-known protective effects of vitamin E against COMT activity [48]. Moreover, we performed epistatic analysis on the two genes which were the most significantly related to WC and susceptibility to AO (*TXN* and *COMT*) using the MDR method, showing that the combination of the two variants (rs2301241 and rs740603) is associated with higher WC and AO than the individual variants. However, how this combination affects AO or fat storage-related mechanisms remains to be tested.

The TXN system is upregulated in high-stress oxidative conditions such as during hypertension episodes (while the GSH system is downregulated [49]), however, this activation still seems insufficient for maintaining a normal redox status in these patients. We hypothesize that high WC, associated with increased OS, increases *TXN* expression and plasma levels of this protein, and therefore T-allele carriers, who have intrinsically lower *TXN* activity, have a higher risk of AO. Moreover, the *TXN* rs2301241 SNP, which is associated with WC, is located between the promoter region and the first intron of this gene which means that it could be involved in gene expression regulation since binding of several transcription factors is extremely sensitive to polymorphisms in regulatory regions. We used Genomatix software [34] to assess the potential functionality of the *TXN* rs2301241 (c.-793T > C) by testing its response to consensus elements in the entire *TXN* promoter. The c.-793C allele site matched a core binding-consensus motif for heat shock transcription factor 2 (*HSF2*) which induces *TXN* expression at the mRNA and protein levels [50]. The fact that *HSF2* can only bind to this reverse region [gtcctgacctcctGAAGtctcagtg] when the c.-793C allele is present indicates the functionality of this polymorphism, and importantly, the differences in *TXN* transcription we describe between groups may be explained by the reduced ability that TT carriers have to produce normal levels of *TXN* mRNA. This correlates with the reduced levels of TXN we found in plasma in T allele carriers, although further studies would be required to further test this hypothesis.

This study should be read and understood within its strengths and limitations. Regarding the former, the age and sex matched cohort we studied was selected from a Spanish region with a very low immigration rate which minimizes error due to population stratification. and the statistics-matching allows to estimate the effect of the outcome without reduced bias due to confounding factors [51], functional study and bioinformatic analysis of the possible effect of *TXN* polymorphism was evaluated. Moreover it is noteworthy that the associations with SNPs were significant and correlated with AO after the adjustment by known confounding variables, and also attached to AO in this study, such as demographic characteristics (including education level), alcohol consumption, biochemical parameters, and presence of other cardiovascular risk factors. In addition, it is important to note that Vitamin E is protective for non-alcoholic fatty liver disease [52]; therefore our data also suggest that subjects with AO should increase their vitamin E intake levels to help with this problem, although this is more important for those with the rs2301241 (*TXN*) TT or the rs740603 (*COMT*) GG genotype. Taking this idea further, by genotyping AO subjects it may be possible to identify a subpopulation of higher-risk allele carriers for which vitamin E supplementation might be beneficial and perhaps protective. However before reaching this conclusion more definitive tests in other groups and populations with different levels of vitamin E intake, as well as studies in different animal models of AO or obesity, will be required; finally, this hypothesis should be tested in prospective studies in order to confirm any effects of increased vitamin E intake.

The main limitations of this study were the absence of a second population to test the replicability of our results, the low reproducibility of the 24-h dietary recall for vitamin intake (despite using validated surveys), and the lack of plasma vitamin level data which we could use to validate reported dietary vitamin intake. Concerning the 24-h dietary recalls, recent articles have specifically shown that the reproducibility of recall by either gender is moderate for vitamin E intake [53]. Moreover, although intake levels and final blood levels are related there are several factors that modify blood levels and there are some major inter-individual variations which can effect plasma levels despite similar initial vitamin intake. Nevertheless, plasma vitamin E concentrations tend to vary little over a wide range of dietary intakes. Kinetic studies with tocopherol suggest that its equilibration into new erythrocytes, liver, and spleen cells is rapid but that turnover in other tissues such as heart, muscle, and adipose tissue is much slower [54]. Another important aspect to consider is the assumption that WC is an indication of abdominal adiposity, although accurate abdominal fat content measurement requires the use of expensive techniques (dual energy X-ray absorptiometry, or whole-body air displacement plethysmography). Therefore the use of WC as a surrogate marker of abdominal fat mass has become an economic, easy, and robust method of obtaining a clinical indication of visceral adiposity. Moreover, it is well established that WC is associated with an increased risk of cardiometabolic syndrome [55] and that it correlates with abdominal fat mass (both subcutaneous and intra-abdominal) [56].

## Conclusions

This study provides the first evidence that genetic variation in OS-related genes can modulate WC in relation to dietary vitamin E intake. Our results indicate the presence of significant associations, in a Spanish population, between two polymorphisms (in the *TXN* and *COMT* genes) and WC, which is influenced by vitamin E intake. In addition, we demonstrate the effect of the rs2301241 polymorphism on *TXN* expression levels, and thus call for further studies to identify the mechanisms linking this gene to WC.

## Additional file

Additional file 1:
**Table S1.** Selected SNP genotyping distribution of WC separately by gender using a dominant genetic model. **Table S2.** *TXN* and *COMT* SNP genotyping distribution of metabolic syndrome using a dominant genetic model. **Table S3.** Metabolic syndrome-related characteristics of the study population categorized by *TXN* and *COMT* SNP genotype. **Table S4.** Correlation analysis between anthropometry and biochemical measurement categorized by *TXN* and *COMT* SNP genotype and case-control.
