# Supplementary material for: The nutrigenetic influence of the interaction between dietary vitamin E and TXN and COMT gene polymorphisms on waist circumference: a case control study
Source: J Transl Med. 2015 Sep 2;13:286. doi: 10.1186/s12967-015-0652-4 (PMC4557824; doi:10.1186/s12967-015-0652-4)
Supplement: Additional file 1: — Table S1. Selected SNP genotyping distribution of WC separately by gender using a dominant genetic model. Table S2. TXN and COMT SNP genotyping distribution of metabolic syndrome using a dominant genetic model. Table S3. Metabolic syndrome-related characteristics of the study population categorized by TXN and COMT SNP genotype. Table S4. Correlation analysis between anthropometry and biochemical measurement categorized by TXN and COMT SNP genotype and case-control. [file 12967_2015_652_MOESM1_ESM.docx]

**Table S1. Selected SNP genotyping distribution of WC separately by gender using a dominant genetic model.**

| **HGN** | **SNP Name** | **Genotype** | **Men** | | **Women** | | **p-value interaction** |
| --- | --- | --- | --- | --- | --- | --- | --- |
|  |  |  | **N** | **WC (cm)** | **N** | **WC (cm)** |  |
| *CAT* | rs1049982 | CC | 133 | 98.22 ±0.78 | 131 | 84.59 ±0.81 | 0.29 |
|  | HWE: 0.17 | CT+TT | 177 | 100.29 ±0.54 | 145 | 85.11 ±0.74 |  |
|  | MAF T: 0.34 |  |  | ns |  | ns |  |
| *SOD3* | rs2536512 | AA | 131 | 99.31 ±0.8 | 119 | 84.03 ±0.85 | 0.24 |
|  | HWE: 0.23 | AG+GG | 178 | 99.44 ±0.54 | 157 | 85.48 ±0.71 |  |
|  | MAF G:0.36 |  |  | ns |  | ns |  |
| *GPX1* | rs3448 | CC | 174 | 98.77 ±0.56 | 166 | 84.15 ±0.68 | 0.80 |
|  | HWE: 0.11 | CT+TT | 135 | 100.25 ±0.75 | 110 | 85.83 ±0.9 |  |
|  | MAF T: 0.25 |  |  | ns |  | ns |  |
| *TXN* | rs4135168 | AA | 177 | 97.88 ±0.57 | 151 | 84.76 ±0.7 | 0.12 |
|  | HWE: 0.61 | AG+GG | 126 | 101.36 ±0.73 | 120 | 85.15 ±0.88 |  |
|  | MAF G:0.24 |  |  | ns |  | ns |  |
| *TXN* | rs2301241 | TT | 88 | 100.79 ±0.89 | 84 | 85.84 ±0.97 | 0.89 |
|  | HWE: 0.24 | TC+CC | 222 | 98.9 ±0.53 | 193 | 84.43 ±0.66 |  |
|  | MAF C:0.47 |  |  | ns |  | ns |  |
| *COMT* | rs740603 | GG | 114 | 100.43 ±0.78 | 111 | 85.37 ±0.88 | 0.52 |
|  | HWE: 0.75 | GA+AA | 193 | 98.65 ±0.56 | 162 | 84.38 ±0.69 |  |
|  | MAF A:0.38 |  |  | ns |  | ns |  |

Values are mean ± SEM. Differences in mean values were assessed by ANOVA and adjusted by age. gender. education level ±<high school. hypertension. glucose. high-density lipoprotein cholesterol. triglycerides. 8-oxodeoxyguanosine and alcohol consumption. HGN: Human Gene Nomenclature.N.: Number of individuals for each genotype or genotype groups; HWE: Hardy-Weinberg Equilibrium; MAF: Minor Allele frequency; WC: Waist circumference; ns: no significant; P-Value interaction denotes differences between SNPs and WC through gender.

**Table S2. *TXN* and *COMT* SNP genotyping distribution of metabolic syndrome using a dominant genetic model.**

| **HGN** | **SNP Name** | **Genotype** | **Non-MS** | **MS** | **OR (95% CI)** | **p-value** |
| --- | --- | --- | --- | --- | --- | --- |
| *TXN* | rs2301241 | TT | 117 (27.1%) | 95 (30.9%) | 1.00 | 0.13 |
|  | HWE: 0.24 | TC+CC | 314 (72.8%) | 212 (69.1%) | 0.69 (0.43-1.12) |  |
| *COMT* | rs740603 | GG | 127 (37.9%) | 93 (43.3%) | 1.00 | 0.062 |
|  | HWE: 0.75 | GA+AA | 208 (62.1%) | 122 (56.7%) | 0.64 (0.40-1.03) |  |

Values are mean ± SEM. Differences in mean values were assessed by ANOVA and adjusted by age. gender. education level ±<high school. hypertension. glucose. high-density lipoprotein cholesterol. triglycerides. 8-oxodeoxyguanosine and alcohol consumption. HGN: Human Gene Nomenclature.MS: Metabolic syndrome; HWE: Hardy-Weinberg Equilibrium;OR: Odds ratio for metabolic syndrome; CI: confidence interval. p-value denotes differences between genotypes using dominant genetic model.

**Table S3. Metabolic syndrome-related characteristics of the study population categorized by *TXN* and *COMT* SNP genotype.**

| **Variables** | ***TXN* (rs2301241)** | | | ***COMT* (rs740603)** | | |
| --- | --- | --- | --- | --- | --- | --- |
|  | **TT (n=172)** | **TC+CC (n=415)** | **p-value** | **GG (n=225)** | **GA+AA (n=335)** | **p-value** |
| Age (yr) | 59 (18) | 62 (17) | ***0.049*** | 59 (17) | 62 (17) | ***0.005*** |
| Weight (Kg) | 73 (13) | 72 (13) | 0.208 | 73 (13) | 72 (12) | 0.103 |
| Height (m) | 163 (9) | 163 (10) | 0.672 | 163 (9) | 162 (9) | 0.110 |
| SBP (mmHg) | 134 (21) | 136 (21) | 0.301 | 135 (21) | 136 (21) | 0.769 |
| DBP (mmHg) | 81 (11) | 81 (10) | 0.434 | 82 (11) | 81 (10) | 0.480 |
| Glucose (mg/dl) | 92 (14) | 92 (14) | 0.926 | 93 (15) | 91 (14) | 0.074 |
| HDL-cholesterol (mg/dl) | 49 (14) | 51 (14) | 0.216 | 50 (14) | 50 (14) | 0.964 |
| Triglycerides (mg/dl) | 183 (86) | 180 (86) | 0.649 | 177 (85) | 183 (86) | 0.393 |

**Table S4. Correlation analysis between anthropometry and biochemical measurement categorized by *TXN* and *COMT* SNP genotype and case-control.**

| **Variables** | | **TXN (rs2301241)** | | | | | |
| --- | --- | --- | --- | --- | --- | --- | --- |
|  |  | **TT** | | | **TC+CC** | | |
|  |  | **Glucose (mg/dL)** | **HDL Cholesterol (mg/dL)** | **Logarithm of triglycerides** | **Glucose (mg/dL)** | **HDL Cholesterol (mg/dL)** | **Logarithm of triglycerides** |
| **Non-AO** | **BMI (kg/m2)** | 0.084 | -0.153 | 0.208* | -0.012 | -0.292** | 0.217** |
|  | **WC (cm)** | **0.236**** | -0.469** | 0.492** | 0.059 | -0.537** | 0.414** |
|  | **Hip (cm)** | 0.107 | -0.013 | 0.114 | -0.007 | -0.192** | 0.145** |
|  | **WHR** | 0.207* | -0.421** | 0.392** | 0.075 | -0.492** | 0.375** |
| **AO** | **BMI (kg/m2)** | -0.061 | -0.231* | 0.043 | -0.122 | -0.199* | 0.189* |
|  | **WC (cm)** | 0.219 | -0.277* | 0.294** | -0.043 | -0.267** | 0.235** |
|  | **Hip (cm)** | -0.025 | -0.307** | 0.042 | -0.223** | -0.127 | -0.038 |
|  | **WHR** | 0.227* | -0.003 | 0.256* | 0.122 | -0.183* | 0.252** |
|  |  |  |  |  |  |  |  |
| **Variables** | | **COMT (rs740603)** | | | | | |
|  |  | **GG** | | | **GA+AA** | | |
|  |  | **Glucose (mg/dL)** | **HDL Cholesterol (mg/dL)** | **Logarithm of triglycerides** | **Glucose (mg/dL)** | **HDL Cholesterol (mg/dL)** | **Logarithm of triglycerides** |
| **Non-AO** | **BMI (kg/m2)** | 0.111 | -0.217** | 0.225** | -0.039 | -0.279** | 0.205** |
|  | **WC (cm)** | **0.191*** | -0.515** | 0.481** | 0.052 | -0.517** | 0.395** |
|  | **Hip (cm)** | 0.091 | -0.080 | 0.105 | -0.027 | -0.188** | 0.159** |
|  | **WHR** | 0.179* | -0.457** | 0.401** | 0.070 | -0.475** | 0.348** |
| **AO** | **BMI (kg/m2)** | -0.027 | -0.116 | 0.110 | -0.173 | -0.296** | 0.173* |
|  | **WC (cm)** | 0.115 | -.265** | 0.216* | -0.008 | -0.288** | 0.284** |
|  | **Hip (cm)** | -0.099 | -0.058 | -0.080 | -0.212* | -0.324** | 0.066 |
|  | **WHR** | 0.217* | -0.232* | 0.298** | 0.130 | -0.043 | 0.211* |

* Statistical significance (p-value< 0.05). **. Statistical significance (p-value< 0.01).
